# Clinical and immunohistochemical analysis of the verrucous and non-verrucous divided nevus of the eyelids

**DOI:** 10.1186/s12886-022-02582-w

**Published:** 2022-09-03

**Authors:** Yuan Deng, Zhengkang Li, Leilei Zhang

**Affiliations:** 1 Department of Ophthalmology, Ninth People’s Hospital, Shanghai JiaoTong University School of Medicine, Shanghai, 200025 P. R. China; 2 Shanghai Key Laboratory of Orbital Diseases and Ocular Oncology, Shanghai, China

**Keywords:** Divided nevus of the eyelids, Verrucous, S100, Ki-67, Melan A, HMB45

## Abstract

**Purpose:**

Divided nevus with verrucous hyperplasia will always recur after surgery but non-verrucous divided eyelid nevus rarely recur. This study analyzed the differential expression of Ki-67, S100, Melan A and HMB45 and identified the correlation between the clinical and histopathological features of verrucous and non-verrucous divided eyelid nevus.

**Methods:**

This study included 29 patients, of whom 8 patients had verrucous divided nevus. Immunohistochemistry labeling was used to assess the expression of Ki-67, S100, Melan A and HMB45 after excision. The difference between verrucous and non-verrucous divided eyelid nevus was analyzed.

**Results:**

The patients’ ages ranged from 2 to 59 years, with a mean age of 19 years. The lesion size ranged from 1.5 to 2.0 cm in diameter and invaded the eyelid margins and the posterior lamella of the eyelids. Immunohistochemistry labeling showed strong positivity for approximately 98.5% of S100 and positive staining for approximately 27.6% of Ki-67, 72.4% of Melan A and 6.8% of HMB45. However, Ki-67 was significantly upregulated in verrucous divided nevus of the eyelids compared with non-verrucous divided nevus, with approximately 38.8% upregulation in verrucous and 18.3% upregulation in non-verrucous nevus.

**Conclusions:**

This study correlated the clinic-pathologic features of verrucous divided eyelid nevus by means of statistically analyzing the varied clinical features and pathological impressions. The significant over-expression of S100 may be used as an indicator of divided nevus of the eyelids, and the over-expressed Ki-67 may contribute to the recurrence of verrucous divided nevus. High expression of HMB45 and Melan A may represent malignant transformation.

## Introduction

Congenital melanocytic nevi occur in approximately 1% of all newborns. Divided nevus is a rare congenital anomaly that was first described in 1919 by Fuchs [1]. In many literatures, it has also been described as “kissing nevus”, “paired nevus”, “panda nevus” or “split ocular nevus”. It occurs on the margins of the upper and lower eyelids of one eye; when the eye is closed, the two nevi look just like one. Due to their neural crest origin, melanoblasts can exhibit localized proliferation [2]. Most of the nevi are flat lesions, pale brown to dark brown without hair; some others are usually slightly elevated, brown or black and pigmented with or without hair, which are defined as verrucous divided nevi of the eyelids. At present, there has been no analysis about verrucous and non-verrucous divided nevus of the eyelids.

The embryonic development, pathogenesis and treatment of divided nevus has rarely been reported in the literature. There has been almost no pathological analysis of divided nevus, as well as no analysis of the difference between verrucous and non-verrucous divided nevus of the eyelids. Ki-67 is a nuclear proliferation marker that is present in all types of tumors. The Ki-67 index has been reported to be higher in malignant melanomas than in benign nevi. Its expression has also been correlated to prognosis in patients with melanomas [3]. S100 has been reported to exhibit high positive rates in malignant melanoma and pigmented disorders (96.7–100%) [4]. In addition, human melanoma black (HMB45) is a monoclonal antibody that was first described in 1986 and recognizes as melanosomal glycoprotein gp100. The anti-gp100 antibody labelling of the cytoplasm of intra-epidermal, “immature” and “activated” melanocytes has a high specificity; its positive expression is only demonstrated in malignant melanoma cells and junctional nevus cells. HMB45 is not expressed in melanocytes in the normal tissue samples around the tumor or in the benign intradermal nevus cells [3, 4]. Moreover, Melan A is a protein antigen that is found on the surface of melanocytes. To date, the expression difference of Ki-67, S100, Melan A and HMB45 has not been detected in divided nevus. In addition, the differential expression of Ki-67, Melan A and HMB45 between verrucous nevi and non-verrucous divided nevi has not yet been investigated.

Therefore, the purpose of this article was to correlate the clinical and the histopathological features of the verrucous and non-verrucous divided nevus of the eyelids. The majority of the divided nevi that were reviewed in this article were the non-verrucous type. In addition, the article suggests that the significant over-expression of S100 could be used as the positive indicator of divided nevus. Moreover, the high expression of Melan A and HMB45 may represent malignant transformation. The over-expressed Ki-67 may contribute to the recurrence of verrucous divided nevus of the eyelids.

## Methods

### Patients and samples

This research followed the tenets of the Declaration of Helsinki. The study was approved by the Institutional Ethics Committee of Shanghai Ninth People’s Hospital and the Ethics Committee of China. The research was performed in accordance with the approved guidelines. Informed consent was obtained from patients prior to participating in the study. Additionally, patient information was collected from Shanghai Ninth People’s Hospital, affiliated with Shanghai JiaoTong University, School of Medicine. The study included 29 cases of divided nevi of the eyelid admitted from 2016 to 2018 (Table 1). The patients were photographed both before and after the operation. 29 tissue samples were obtained from patients in the Department of Ophthalmology at the Shanghai Ninth People’s Hospital (Shanghai, China). The patients were 2–59 years old (mean age:19 years; 14 males and 15 females) and provided written informed consent. The follow-up time period was 52 months. There were 4 patients recurred and all of them were verrucous nevus. The average recurrence time was 5 months. The paraffin-embedded tissues were sectioned (size: 5 μm) and placed on the prepared slides and dried at 37 °C overnight.

**Table 1:** Clinical characteristics of divided nevus of the eyelid patients from 2016 to 2018

	Sex	Age	Type	Size	Recurrence	Classification	Follow-up Time (months)
1	F	22	intradermal nevus	small	no	Non-verrucous	49
2	M	17	intradermal nevus	small	no	Non-verrucous	50
3	F	33	mixed nevus	medium	yes	Verrucous	50
4	M	2	junctional nevus	small	no	Non-verrucous	50
5	M	22	intradermal nevus	small	no	Non-verrucous	51
6	F	31	intradermal nevus	small	no	Verrucous	51
7	F	59	intradermal nevus	small	no	Non-verrucous	52
8	F	22	intradermal nevus	small	no	Verrucous	52
9	M	12	mixed nevus	small	no	Verrucous	53
10	M	17	mixed nevus	small	yes	Verrucous	60
11	F	28	intradermal nevus	small	no	Non-verrucous	52
12	F	19	mixed nevus	medium	no	Non-verrucous	53
13	F	7	intradermal nevus	small	no	Non-verrucous	53
14	M	10	mixed nevus	small	no	Non-verrucous	53
15	F	7	intradermal nevus	small	yes	Verrucous	61
16	M	12	mixed nevus	small	no	Non-verrucous	62
17	M	23	junctional nevus	large	no	Verrucous	60
18	F	12	intradermal nevus	small	no	Non-verrucous	66
19	F	21	junctional nevus	small	no	Non-verrucous	64
20	M	23	intradermal nevus	medium	no	Non-verrucous	60
21	F	37	intradermal nevus	small	no	Non-verrucous	42
22	M	5	mixed nevus	small	no	Non-verrucous	41
23	M	6	mixed nevus	small	no	Non-verrucous	61
24	F	6	junctional nevus	large	no	Non-verrucous	41
25	M	33	intradermal nevus	small	no	Non-verrucous	60
26	F	26	mixed nevus	large	yes	Verrucous	44
27	M	25	intradermal nevus	large	no	Non-verrucous	42
28	F	45	intradermal nevus	large	no	Non-verrucous	40
29	M	18	mixed nevus	medium	no	Non-verrucous	37

### HE and immunostaining

The specimens obtained from patients undergoing surgery were cut into two pieces and then fixed with formalin for 24 h. The myocutaneous sliding flap was used for the restructuring the eyelid of divided nevus [5]. The next day, the formalin-fixed tissues were embedded in paraffin to generate formalin-fixed, paraffin-embedded (FFPE) tissues, which were cut into 5-μm-thick sections. Paraffin-embedded tissue sections were stained via a simple simultaneous IHC double staining technique according to the instructions. The samples were incubated at 37 °C for 10 min with 3% hydrogen peroxide and then washed three times for 2 min in PBS. The samples were incubated at 37 °C for 2 h with primary antibodies against S100 (1:250, clone: EPR5250), HMB45 (1:250, clone: EP4863(2)), Ki-67 (1:250, clone: EPR3610) and Melan A (1:250, clone: EPR20380). After being washed in PBS three times, the coverslips were incubated in fluorescently conjugated secondary antibodies (Alexa Fluor 546 goat anti-mouse or goat anti-rabbit, 1:800 in PBS, BD) for one hour at room temperature. For all of the samples that contained melanin, we chose the red staining and safranin O staining. Therefore, the specificity index is marked in red. Both simple semiquantitative estimates of the immunopositivity in the deepest third of the lesions and full-scale quantitative measurements of the Ki-67 and HMB45 indices were performed, and scores for melanomas and nevi were compared. At least three fields from each section were observed under the microscope. Cells with granules in the cytoplasm or nucleus were considered to be positive for S100 protein and HMB45, Melan A and Ki-67 [6]. The immunoreactive sections were visualized and imaged using a fluorescent microscope (Olympus BX51, Japan). The staining intensity of S100 protein and HMB45, Melan A and Ki-67 was analyzed via Image-Pro Plus 6.0 software. Three sections from different parts were taken the histological analyses.

## Results

### Eyelids divided nevus compound of verrucous nevus and non-verrucous nevus

**Fig. 1:**
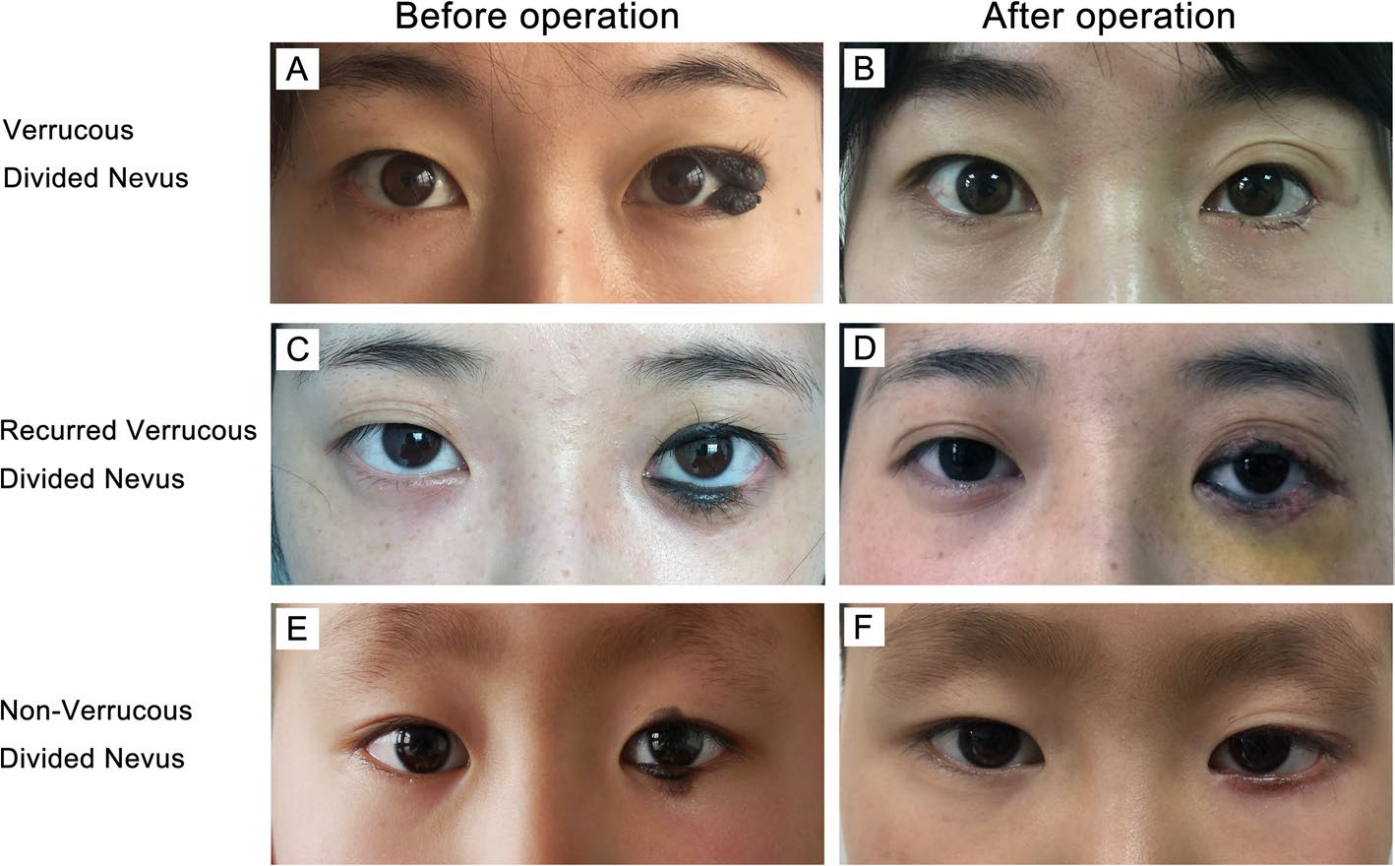
Typical photos of divided nevus of the eyelids. **A**, **B** The verrucous divided nevus was black with papillary protuberances and hair located on the left upper and lower eyelid margins before and after the operation. **C**, **D** The recurrent verrucous divided nevus was black with papillary protuberances and hair located on the left eyelid margin before and after the operation. **E**, **F** The non-verrucous divided nevus was dark brown without papillary protuberances, and some hair was located on the right eyelid margin

**Fig. 2:**
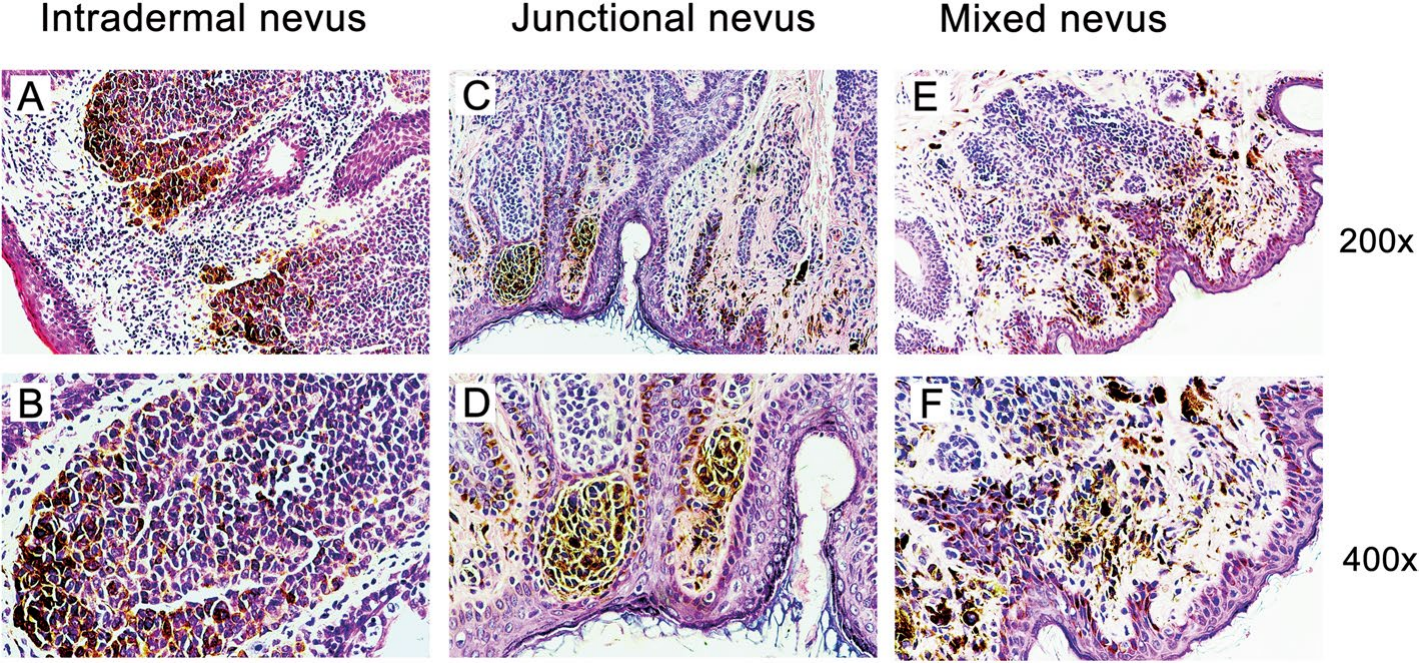
The pathological features of divided eyelid nevus tissues stained with HE staining. (H&E, original magnification 200 X and 400 X, respectively). Typical histology of divided eyelid nevus tissues was pictured as intradermal nevus (**A** and **B**), junctional nevus (**C** and **D**) and mixed nevus (**E** and **F**)

The patients with divided nevus eyelid aged from 2 to 59 years, with an average age of 19 years (Table 1). However, the symptoms appeared after birth. They were different in size, shape and distribution. They occurred on the upper and the lower eyelids, with or without the inner canthus. Additionally, the color varied from brown to black and some were covered with hair (Fig. 1). The lesions ranged from 0.2 to 3.0 cm in diameter and invaded the eyelid margins and the posterior lamella of the eyelids. Small cases (< 1.5 cm) accounted for the majority of the patients (approximately about 70.0%), whereas medium, cases (1.5–2.0 cm) accounted for 13.8% of the patients, and large cases (> 2.0 cm) accounted for 17.2%. Of all the 29 patients observed, there were 15 intradermal nevus (51.7%), 10 mixed nevus (34.5%) and 4 junctional nevus (13.8%) cases (Fig. 2). The verrucous nevi were observed in 8 cases (27.6%), and non-verrucous nevi were observed in 21 cases (62.4%). According to the different locations of nevus cells on the skin, verrucous nevus included 4 intradermal nevus (50.0%), 3 mixed nevus (37.5%) and 1 junctional nevus (12.5%). Non-verrucous nevus included 11 intradermal nevus (52.4%), 7 mixed nevus (33.3%) and 3 junctional nevus (14.3%). In addition, the intradermal nevus accounted for the most divided nevus. Verrucous nevus protruded on the skin surface with or without melanin. After surgery, 4 patients recurred with an average recurrence time was 5 months. It is noteworthy that all of the 4 patients had verrucous nevus. The recurrence rate was 50% in verrucous nevus patients, whereas there was no recurrence in non-verrucous nevi after surgery. This finding demonstrated that the verrucous nevus was more likely to recur than non-verrucous nevus. In addition, the melanin was found using HE detection. The recurrence of verrucous nevus involves the eyelid in a wide range, in which three recurrences are large and one is medium in size. Finally, due to the wide involvement of the eyelid, the remaining nevus cells may proliferate under the drive of high expression of Ki-67. However, due to the limited cases, further study and analysis are required.

### Differential expression of S100, Ki-67, Melan A and HMB45

**Fig. 3:**
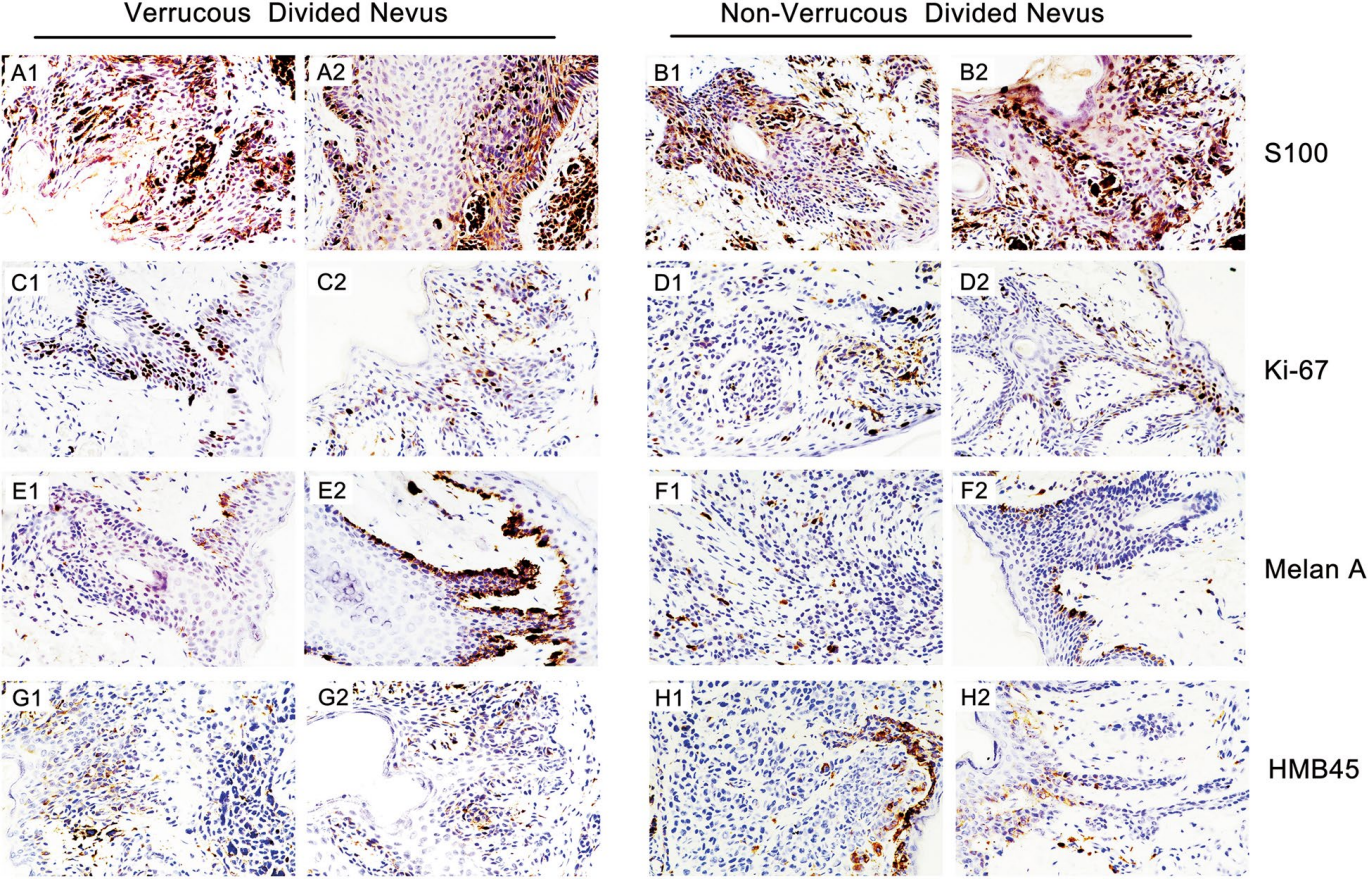
The expression of S100, Ki-67, Melan A and HMB45 in divided eyelid nevus tissues (400 X). The S100 expression was approximately 98.5% (**A1**, **A2** and **B1**, **B2**). The expression of Ki-67 was approximately 38.8% (**C1** and **C2**) in verrucous nevi and 18.3% (**D1** and **D2**) in non-verrucous nevus. The expression of Melan A was approximately 72.4% in divided nevi, with an average rate of approximately 78.6% (**E1** and **E2**) in verrucous nevi and approximately 55.1% (**F1** and **F2**) in non-verrucous nevus without a significant statistical difference. The average expression of HMB45 was approximately 6.8%, with an average rate of approximately 9.2% (**G1** and **G2**) in verrucous and approximately 3.8% (**H1** and **H2**) in non-verrucous nevus without a significant statistical difference. Representative IHC images are shown here

The expression of Ki-67, S100, Melan A and HMB45 was detected using IHC staining. There were high positive rates of S100 (approximately 98.5%), Ki-67 (approximately 27.6%) and Melan A (approximately 72.4%); however, the positivity rate of HMB45 was quite low (6.8%). The results demonstrated a high expression of S100 in approximately 98–100% of the divided nevi. Additionally, the expression of Ki-67 was approximately 7.7–67.0%, with an average percent of 27.6%. However, the expression of Ki-67 was significantly upregulated in verrucous divided nevus of the eyelids compared with the divided nevus without verrucous hyperplasia, with an average rate of approximately 38.8% in verrucous and approximately 18.3% in non-verrucous nevus, with a significant statistical difference (*P* < 0.05). Additionally, Melan-A is a protein antigen that is found on the surface of melanocytes. From our results, there were approximately 72.4% in divided nevus, with an average rate of approximately 78.6% in verrucous nevi and approximately 55.1% in non-verrucous nevus without significant statistical difference. HMB45 immunostaining has also been considered as a helpful tool to distinguish benign from malignant melanocytic tumors. Nevertheless, HMB45 immunostaining identified epidermis in Spitz nevus, but no positive reactions were found. The expression of HMB45 was almost negative in the divided nevus of eyelid. Furthermore, the average expression of HMB45 was approximately 6.8%, with an average rate of about 9.2% in verrucous and approximately 3.8% in non-verrucous nevus without significant statistical difference (Fig. 3).

## Discussion

Divided nevus is a type of congenital nevus that affects the opposing of the upper and lower eyelids. It is thought to originate from melanocyte migration or Schwann cells of a neuroectodermic origin during the embryological fusion of the lids, thus producing the “kissing” nevus [7]. By the 9th to 10th gestational week (38–40 mm) the two eyelids meet and the epidermal layer fuses, without any connecting mesenchymal layer. During the 20th week, the eyelids begin to separate, after lipids start to appear at the junctional zone, with complete separation occurring at 28 to 30 weeks (180 to 200 mm) [2]. Herein, the median age of patients was 19 years, but the nevi had been observed since birth. Similar lesions have been reported in other parts of the body, such as phalanges and penis [8].

Two classification systems have typically been used to describe congenital nevi based either on total size or on histology. On the basis of size classification, they are categorized as a) small (< 1.5 cm), b) medium (1.5–2.0 cm), and c) large (> 2.0 cm). In our cases, small cases accounted for the majority of the cases with about 70.0%, whereas medium cases accounted for 13.8% and large cases accounted for 17.2%. In addition, according to the different locations of nevus cells in the skin, divided nevus can be divided into three types: junctional nevus, mixed nevus and intradermal nevus. In this study, 15 intradermal nevus (51.7%), 10 mixed nevus (34.5%) and 4 junctional nevus (13.8%) were observed. Verrucous nevus included 4 intradermal nevus (50.0%), 3 mixed nevus (37.5%) and 1 junctional nevus (12.5%). Non-verrucous nevus included 11 intradermal nevus (52.4%), 7 mixed nevus (33.3%) and 3 junctional nevus (14.3%). Specifically, intradermal nevus accounted for the most divided nevus cases.

Divided nevus usually increases with age. Excision with or without skin grafting is a common method for treating divided nevus of the eyelid. Numerous methods for reconstruction have been described, including the entire reconstructive ladder with both one- and two-stage approaches according to the size of the divided nevus [9]. The procedures mentioned in the literature include primary closure, local or distant tissue flaps (such as the skin graft ranging widely from the postauricular, contralateral eyelid, upper arm, and supraclavicular region, etc.) and mucous membranes (nasal and oral membranes, among others) [5, 10–12]. In addition, other types of methods such as dermabrasion [13], cryotherapy [14] and CO_2_ laser [15], have been used. Regardless, the ultimate treatment should be individualized and dependent on the size, type and the extent of involvement of the tumors. In the follow-up, compared with a divided nevus without verrucous hyperplasia, a verrucous divided nevus of the eyelids was more likely to recur 1–2 years after surgery. In this study, 4 verrucous patients experienced recurrence, accounting accounted for 50% of the patients, which demonstrated that the verrucous nevus nevi were more likely to recur than non-verrucous nevus. However, the hyperplastic state of the verrucous divided eyelid nevus was not discussed. This study explored the characteristics of recurrent verrucous nevus.

Subsequently, the expression of S100, Ki-67, Melan A and HMB45 and different expressions between verrucous and non-verrucous divided nevus of the eyelids were analyzed. S100 typically exhibits high positive rates in malignant melanoma and pigmented disorders. Herein, the expression of S100 was approximately 98.5% in divided eyelid nevus, but there was no difference between verrucous (98.8%) and non-verrucous divided nevus (98.0%). Ki-67 is a nuclear proliferation marker that is present in all types of tumors and usually indicates the active proliferation of cells. It has been widely used in the diagnosis of the malignant potential behaviors of tumors [16]. Different thresholds have been evaluated in an attempt to identify a cutoff between nevi and melanoma, with final proposed cutoffs of 2–10% being identified. In this study, the average positive rate of Ki-67 was approximately 27.6% in divided nevus. However, we observed 38.8% expression of Ki-67 in verrucous divided nevus versus and 18.3% expression in non-verrucous divided nevus. Specifically, verrucous nevus exhibits a stronger proliferative ability. However, there was a report indicating that Ki-67 was useless in predicting the risk of malignant transformation of small or medium size congenital nevus [17]. In our report, the average age of the 4 recurrences at visit was 19 years, which were observed after birth. The recurrence of verrucous nevus involves the eyelid over a wide range, in which three recurrences were large in size and one recurrence was medium in size. Finally, due to the wide involvement of the eyelid, the remaining nevus cells may proliferate under the drive of the high expression of Ki-67. However, the number of cases is limited, and more cases are needed for statistical analysis in the future.

Antibodies against the antigen are used in the medical specialty of anatomic pathology in order to recognize cells of melanocytic differentiation, which is useful for the diagnosis of a melanoma. HMB45 immunostaining has also been considered as a helpful tool to distinguish benign from malignant melanocytic tumors. It has been reported that the positive rate of HMB45 in primary melanoma is 69–93% [18]. HMB45 and Melan A expression has been demonstrated in endometrial stromal sarcomas [19]. In our cases, the expression of Melan A was approximately 72.4% and HMB45 was only approximately 6.8%. To date, there have been no documented cases of malignant transformation or malignancy in any of the divided lesions observed in the literature review [20]. In addition, the vast majority of the divided nevi indicated in this review were of the melanocytic intradermal type. Junctional activity, or nevus cells present at the epidermal–dermal junction, has been observed in some of the lesions. However, there was no significant difference between verrucous and non-verrucous divided nevus of the eyelids.

## Conclusions

The results of this study indicate that over-expressed Ki-67 may be associated with the recurrence of verrucous divided nevus of the eyelid, whereas the expression of S100, Melan A and HMB45 was not significantly different. The significant over-expression of S100 may be used as an indicator of divided nevus of the eyelids, and the over-expressed Ki-67 may contribute to the recurrence of verrucous divided nevus. High expression of HMB45 and Melan A may represent malignant transformation. Due to the limited number of cases, further studies are needed to confirm these results.

### Statistical analysis

The Mann–Whitney U test was used for the analysis of IHC distribution. The Chi-square test was used to analyze the difference between groups recurrence and non-recurrence groups in verrucous nevus and non-verrucous nevus via SPSS19.0 software. All of the experiments were performed three times. A value of *p* < 0.05 was considered to be statistically significant.

## Data Availability

The datasets analyzed during this study are not publicly available due to the data is too large to upload, but are available from the corresponding author on reasonable request.

## References

[1] Fuchs A. Divided nevi of the skin of the eyelid. Klin Monatsbl Augenheilkd. 1919;63:678. 13702351

[2] Hamming N (1983). Anatomy and embryology of the eyelids: a review with special reference to the development of divided nevi. Pediatr Dermatol.

[3] Gown AM, Vogel AM, Hoak D, Gough F, McNutt MA (1986). Monoclonal antibodies specific for melanocytic tumors distinguish subpopulations of melanocytes. Am J Pathol.

[4] Xia J, Wang Y, Li F, Wang J, Mu Y, Mei X, Li X, Zhu W, Jin X, Yu K (2016). Expression of microphthalmia transcription factor, S100 protein, and HMB-45 in malignant melanoma and pigmented nevi. Biomed Rep.

[5] Tang W, Zhang L, Li Z, Deng Y (2021). Myocutaneous sliding flap for reconstruction of divided eyelid nevus. Can J Ophthalmol.

[6] Hague A, Moorghen M, Hicks D, Chapman M, Paraskeva C (1994). BCL-2 expression in human colorectal adenomas and carcinomas. Oncogene.

[7] Andersen H, Ehlers N, Matthiessen ME (1965). Histochemistry and development of the human eyelids. Acta Ophthalmol (Copenh).

[8] Yun SJ, Wi HS, Lee JB, Kim SJ, Won YH, Lee SC (2011). Kissing nevus of the penis. Ann Dermatol.

[9] Rajput GC, Mahajan D, Chaudhary KP, Deewana V (2015). Kissing naevus arising from neural crest cells presenting as upper and the lower lid mass. J Neurosci Rural Pract.

[10] Jia R, Zhu H, Lin M, Li Z, Sun Y, Luo M, Fan X (2012). Clinicopathological characteristics and surgical outcomes of divided nevus of the eyelids: a decade's experience on 73 cases. Ann Plast Surg.

[11] Cho HJ, Lee W, Jeon MK, Park JO, Yang EJ (2019). Staged mosaic punching excision of a kissing nevus on the Eyelid. Aesthetic Plast Surg.

[12] Liu J, Sun J, Wang Z, Guo L, Guo N (2020). Treatment of divided eyelid nevus with orbicularis oculi myocutaneous flap: report of 17 cases. Ann Plast Surg.

[13] Miller CJ, Becker DW (1979). Removing pigmentation by dermabrading naevi in infancy. Br J Plast Surg.

[14] Ehlers N (1969). Divided nevus. Acta Ophthalmol (Copenh).

[15] Zeng Y (2014). Divided nevus of the eyelid: successful treatment with CO2 laser. J Dermatolog Treat.

[16] Menon SS, Guruvayoorappan C, Sakthivel KM, Rasmi RR (2019). Ki-67 protein as a tumour proliferation marker. Clin Chim Acta.

[17] Lejeune C, Laporte M, Musette S, Petein M, Heenen M (2009). Interest of immunohistochemic markers (Ki67, HMB45, p53) in risk analysis of congenital naevi of little and middle size. Rev Med Brux.

[18] Ohsie SJ, Sarantopoulos GP, Cochran AJ, Binder SW (2008). Immunohistochemical characteristics of melanoma. J Cutan Pathol.

[19] Albores-Saavedra J, Dorantes-Heredia R, Chable-Montero F, Chanona-Vilchis J, Perez-Montiel D, Lino-Silva LS, Gonzalez-Romo MA, Ramirez-Jaramillo JM, Henson DE (2014). Endometrial stromal sarcomas: immunoprofile with emphasis on HMB45 reactivity. Am J Clin Pathol.

[20] Desai SC, Walen S, Holds JB, Branham G (2013). Divided nevus of the eyelid: review of embryology, pathology and treatment. Am J Otolaryngol.

